# A fully labelled image dataset of banana leaves deficient in nutrients

**DOI:** 10.1016/j.dib.2023.109155

**Published:** 2023-04-18

**Authors:** Sunitha P, Uma B, Channakeshava S, Suresh Babu C S

**Affiliations:** a Department of Computer Science and Engineering, Malnad College of Engineering, Hassan, Karnataka, India; b Department of Soil Science, Agriculture College, Tarekere, Hassan, Karnataka, India; c Department of Electronics and Instrumentation Engineering, Malnad College of Engineering, Hassan, Karnataka, India

**Keywords:** Nutrient, Processing, Machine learning, Deficiency

## Abstract

Agriculture is the art and science of cultivating the soil, growing crops and raising livestock. Enhancing crop yield becomes essential to improve economy in agriculture sector. Crops need 16 essential nutrients in balanced factor for their proper growth. Deficiency among these essential nutrients causes stunted growth often leading to significant crop loss. Symptoms of nutrient deficiency in plants can be observed visually which needs to be diagnosed correctly to rectify the problem, so that plants can grow healthily, increasing its yield.

Among various crops, banana is one of the staple foods for millions of people across country and world. They contain essential nutrients that can have a defending impact on health of the human beings. The year-round availability, affordability, varietal range, taste, nutritive of banana and medicinal value makes it the favorite fruit among all classes of people. In addition, it also has good export potential. Few of the symptoms due to nutrient's deficiency in banana leaves are like curling of leaves, appearance of yellow strips, yellowing of the leaves, bluish color of young leaves. Deficiency symptoms can be visualized prominently on the leaves of the plant. Further, Machine Learning models can be developed to detect nutrient deficiency in leaves and help farmers in taking relevant measures. Thus, a fully labelled dataset becomes essential to train and test these models to detect nutrient deficiency accurately.

The dataset created consists of banana leaf images of various categories like Musa acuminata (Dwarf Cavendish), Robusta, Rasthali, Poovan, Monthan, Elakkibale. Images depict deficiency in eight class of nutrients: boron, calcium, iron, potassium, manganese, magnesium, sulphur and zinc. Table 1 summarizes the essential nutrients and their deficiency symptoms visible on the leaves. Dataset also contains images of healthy leaves. Machine Learning Models can be developed by researchers and students and train them by using the created banana dataset to obtain high accuracy.

**Specifications Table**

**Table:** 

Subject	Agriculture Engineering
Specific subject area	Machine Learning, Deep Learning
Type of data	• Images of banana leaves which are deficient in nutrients – boron, calcium, potassium, iron, magnesium, manganese, sulphur, zinc. • Healthy leaf images are also included into the dataset.
How the data were acquired	Nutrient deficient banana leaves are captured using mobile phone camera. To collect versatile dataset, various mobiles with varying camera resolution are being used to capture images. Few mobiles used are: Samsung M20, A12 having camera resolution of 13MP and 48 MP, Redmi with 40 Mega Pixel, Motorola G5 and G5PLUS with 14Mp and 48MP. Images are taken under natural morning light as well as evening light.
Data format	Raw images captured are in the format of .jpg and .png
Description of data collection	Banana leaf images are captured using mobiles with high resolution cameras. Images were taken in the morning as well as in the evening sessions to obtain images in various environmental conditions. Banana plantations were visited manually to get images of nutrient deficient leaves. Samples are being labelled by an agriculture expert. Original dimension of the images was 3072 × 4096, 3096 × 4128, 3000 × 4000. All images are being resized to a standard size of 225 × 225. Natural background of the images is being removed and black background is set to black to maintain proportions of the images in the dataset [8]. The input image size chosen for existing pre-trained deep learning models VGG16, ResNet, Inception V3 is 224 × 224 pixels [8]. The original dimension of the image is 3072 × 4096, 3096 × 4128, 3000 × 4000, but, the computational speed of the models increases with increase in the size of the input image. Hence, the images are resized to 225 × 225 using Adobe Photoshop tool to reduce the computational speed. The tool resizes the images without changing the quality of the images.
Data source location	• District Hassan ○ Nittur, Tamlapura, Saligrama, Tarekere • District Ramnagara ○ Garkalli • District Shimoga ○ Bhadravathi • District Davangere ○ Maganahalli, Channagiri • District Hosapete ○ Hosuru All districts are in Karnataka, India.
Data accessibility	Repository name: “Images of Nutrient Deficient Banana Plant Leaves”, Mendeley Data, V1 Data identification number: DOI: doi:10.17632/7vpdrbdkd4.1 Direct URL to data: https://data.mendeley.com/datasets/7vpdrbdkd4

## Value of the Data

Artificial Intelligence has taken over every field in the world - Marketing, Agriculture, Industry, Finance, Technology, etc. Machine Learning and Deep Learning models need to be trained with huge amount of data to obtain high precision results [1], [2]. Thus, these models are data hungry.

• The dataset of nutrient deficient banana leaves contains images belonging to eight class of nutrients.
• The dataset provides raw data of 3000 images and is augmented to 7000+ images of eight classes.
• The images are manually labelled by an agriculture scientist.
• The present comprehensive dataset can help researchers / students to evaluate their Machine / Deep Learning models and can achieve high precision results.

## 1 Objective

• Create image dataset of banana leaves which are deficient in nutrients.
• Collect images of various category of banana plants across different locations.
• Gather diverse data by capturing images of the leaves from various angles, different perspectives and in variable lighting conditions.

## 2 Data Description

Banana is a rich source of carbohydrate and is rich in vitamins particularly vitamin B. The fruit is easy to digest, free from fat and cholesterol. It helps in reducing risk of heart diseases when used regularly and is recommended for patients suffering from high blood pressure, arthritis, ulcer, gastroenteritis and kidney disorders. Banana is considered a foodie or heavy feeder plant, so soil fertility is really important for it [3].

Banana is a quick growing and short-lived plant. It responds very well to the application of balanced nutrients. Deficiency of the essential nutrients leads to stunted growth of the plants thus decreasing their yield. Symptoms of deficiency in nutrients can be prominently seen on the leaves of the plants [4], [5]. Machine Learning models can be designed to detect the deficiency symptoms by processing the images of the leaves. Hence, dataset of leaf images which are deficient in nutrients become essential to train Machine learning models.

The dataset created consists of images of banana leaves which are deficient in nutrients. Images captured belong to various categories of banana across different locations in Karnataka, India. The dataset contains eight folders labelled with each nutrient class namely: Boron, Calcium, Iron, Potassium, Magnesium, Manganese, Sulphur and Zinc. Table 2 tabulates the sample of images of each nutrient category with their deficiency symptoms [5]. In addition, healthy leaves are stored in another folder. Leaves can also be found to be deficient in multiple nutrients [6]. Hence, more than one folder can contain same leaf images. The captured raw images are processed by resizing it to a standard size of 225 × 225. Images are cropped manually to remove unwanted parts and background is set to black so that designed Machine learning models get trained with uniform images and report high accuracy [2]. In addition, the accuracy of the Machine/ Deep Learning model depends on the richness of the dataset [6], [7]. Hence, the created dataset containing 3000 raw images, is augmented to 7000+ images. Augmentation is carried out in such a manner that each category has approximately same number of images, so that Machine /Deep Learning models should not undergo training with imbalanced dataset.

(see Table 1)

**Table 1:** Deficiency of nutrients and their symptoms.

Sl. No	Nutrient	Deficiency Symptoms
1.	Potassium	Orange Yellow color in old leaves, curling of midribs
2.	Boron	Reduced leaf area, curling of leaves, lamina deformation, appearance of white stripes perpendicular to the veins on the lamina of young leaves
3.	Magnesium	Yellow discolouration is observed in the mid blade and midrib portion, the margins of the leaf remain green. Purple mottling of the petioles, Leaf symptoms include marginal yellowing
4.	Sulphur	Yellow or white appearance of young leaves, necrotic patches on the leaf margins, leaves take on a silver-green colouring
5.	Iron	Younger leaves become pale green or white in colour
6.	Zinc	Anthocyanin pigmentation appear on its underside, yellow to white stripes between the secondary veins, alternating yellow and green stripes
7.	Manganese	Narrow green edge appears at the leaf margins of second or third youngest leaf, which further spreads along the main veins towards the midrib
8.	Calcium	Youngest leaf become abnormal in size, leaf margins become dry and veins become erupted and cause the spike leaf

**Table 2:** The sample banana leaf images which are deficient in nutrients of 8 categories.

## 3 Experimental Design, Materials and Methods

### 3.1 Design and Materials

Mobile camera is used to capture banana leaf images to create dataset of nutrient deficiency. Mobile cameras of Samsung M20, A12, Motorola G5, ONEPlus, RedMi, were used to capture images. Images are captured in different environmental conditions like in the morning and evening. Few images were also taken in the afternoon due to which images have shaded regions. Certain Images also contain dew drops as they were taken in the morning time. Images are acquired from various locations in Karnataka, India.

### 3.2 Methods

Raw images are collected from various banana plantations of different categories in several places of Karnataka state, India. Plantations were visited manually and images were taken under different sunlight conditions using mobile camera. Images are stored in in .jpg and .png format. Images are annotated with respect to eight class of nutrients by an agriculture expert and are stored in eight separate directories respectively. These images are being processed to remove background using on line tool remove.bg. Further, to remove the unwanted regions, images are being cropped manually using photoeditor. All images are being resized to 225 × 225 and background is set to black so that Machine Learning algorithms get uniform images to train the model [2].

Dataset is expanded to 7000+ images through data augmentation in order to improve the performance and ability of the Machine Learning model. Augmentation is carried out to obtain uniform distribution of images among all class of nutrients. Table 3 gives detail specification of the camera and quality of the images. Table 4 gives information about the type of banana in various locations where in the images are captured. Table 5 depicts the botanical/scientific names of various categories of banana of the image dataset. Table 6 summarizes the total number of raw and augmented images of each category of nutrients and Table 7 gives the count of healthy leaves of banana dataset.

**Table 3:** Specifications of the camera and the Images captured.

Sl. No	Camera Specifications	Samsung M20	Samsung A12	Moto g5	Moto g5+	RedMI,	ONEPlus 7 Pro
1.	Mega Pixel	14	48	14	48	48	48
2.	Dimension	1080 × 2440	730 × 1600	1080 × 2400	2080 × 1920	1080 × 2400	1440 × 3120
3.	Horizontal Resolution	1080	730	1080	2080	1080	1440
4.	Vertical Resolution	2440	1600	2400	2080	2400	3120
5.	Bit Depth	96dpi	96dpi	96dpi	96dpi	96dpi	96dpi

**Table 4:** Description of the types of bananas at several locations.

Sl. No	Plantation Location	Type of Banana	Deficiency
1.	Bhadravathi	Puttabale	Potassium, Sulphur, Zinc
2.	Chennagiri	Puttabale	Calcium, Iron, Magnesium, Zinc
3.	Davangere	Puttabale	Calcium, Magnesium, Potassium, Sulphur, Zinc
4.	Garkalli	ElakkiBale	Boron, Calcium, Magnesium, Potassium, Sulphur, Zinc
5.	Nittur	Puttabale, Pach Bable	Boron, Magnesium, Zinc
6.	Hosapete	Elalakki, Sugandhi,Sakkare	Potassium, Calcium
7.	Shanthinagar	Puttabale, Pach Bable	Boron, Magnesium, Zinc
8.	Tarekere	Puttabale	Boron, Magnesium, Zinc
9.	Tamlapura	Pachbale	Magnesium, Potassium, Zinc

**Table 5:** The botanical/scientific names of different categories of banana.

Sl. No	Type of Banana	Scientific Name
1.	Puttabale, ElakkiBale	Musa Balbisiana
2.	Pach Bable	Musa Accuminata
3.	Sugandhi, Sakkare	Musa Paradisiaca

**Table 6:** Description and count of images belonging to eight class of nutrients.

Sl. No	Nutrient	No. of Samples	Augmented number of samples
1.	Boron	100	800
2.	Calcium	440	880
3.	Iron	86	860
4.	Magnesium	160	800
5.	Manganese	24	840
6.	Potassium	210	840
7.	Sulphur	730	730
8.	Zinc	400	800
9.	Total	3000	6550

**Table 7:** Count of healthy leaf images.

Sl. No	Leaves type	No. of samples	Augmented number of samples
1.	Healthy	950	950

## Ethics Statements

The annotated image dataset is original and is not published elsewhere. No funding nor financial support is provided for the present work. There is no conflict of interest. The dataset is made available for public.

## CRediT authorship contribution statement

**Sunitha P:** Data curation, Formal analysis, Methodology, Resources, Software, Validation, Writing – original draft, Writing – review & editing. **Uma B:** Formal analysis, Investigation, Methodology, Project administration, Resources, Supervision, Visualization, Writing – review & editing. **Channakeshava S:** Conceptualization, Data curation, Supervision. **Suresh Babu C S:** Resources, Writing – review & editing.

## Declaration of Competing Interest

The authors declare that they have no known competing financial interests or personal relationships that could have appeared to influence the work reported in this paper.

## Data Availability

Images of Nutrient Deficient Banana Plant Leaves (Original data) (Mendeley Data).
